# Effects of Novel Dinuclear Cisplatinum(II) Complexes on the Electrical Properties of Human Molt-4 Leukemia Cells

**DOI:** 10.1007/s12013-014-0375-9

**Published:** 2014-11-16

**Authors:** Izabela Dobrzyńska, Elżbieta Skrzydlewska, Zbigniew A. Figaszewski

**Affiliations:** 1 Institute of Chemistry, University in Białystok, Al. Piłsudskiego 11/4, 15-443 Białystok, Poland; 2 Department of Analytical Chemistry, Medical University of Białystok, Mickiewicza 2, 15-230 Białystok, Poland; 3 Laboratory of Electrochemical Power Sources, Faculty of Chemistry, University of Warsaw, Pasteur St. 1, 02-093 Warsaw, Poland

**Keywords:** Surface charge density, Leukemia cells (Molt-4), Lipid peroxidation

## Abstract

The aim of this study was to determine the influence of cisplatin and novel dinuclear platinum(II) complexes on the membrane electrical properties and lipid peroxidation levels of the Molt-4 human leukemia cell line. Changes in cell function may affect the basal electrical surface properties of cell membranes. These changes can be detected using electrokinetic measurements. Surface charge densities of Molt-4 cells were measured as a function of pH. A four-component equilibrium model was used to describe the interaction between the ions in solution and on cell membrane surfaces. Agreement was found between the experimental and theoretical charge variation curves of the leukemia cells at pH 2.5–9. Lipid peroxidation was estimated by measuring levels of 8-iso-prostaglandine F2α [isoprostanes]. Acid and base functional group concentrations and average association constants with hydroxyl ions were smaller in cisplatin- or dinuclear platinum(II) complex-treated leukemia cell membranes compared to those in untreated cancer cells, and the average association constants with hydrogen ions were higher. Levels of lipid peroxidation products in cisplatin- or dinuclear platinum(II) complex-treated leukemia cell were higher than those found in untreated cancer cells.

## Introduction

Hematopoietic cells exist in a state of proliferation, differentiation, or apoptosis. Under normal circumstances, hematopoietic cell proliferation and apoptosis are carefully balanced. Induction of differentiation is associated with a loss of proliferative capacity, and cell death accompanies hematopoietic cell maturation. Leukemic transformations can be related to dysregulation of any of these processes. Considerable evidence supports the notion that leukemias are likely to arise from disruption of hematopoietic progenitor differentiation as well as diminished ability to undergo apoptosis [7].

In leukemic cells, ultrastructural architecture changes in the plasmalemma result in alterations of the biologic properties. Any perturbations in the actions of the cell function are manifested by variations in the actions of the electric bilayer. An essential property of the electric bilayer is its electrical charge, which can be altered by cancerous transformation or various drugs. For these reasons, studies focused on membrane electrical charge can provide information on the equilibrium within a membrane and between the membrane and its environment, under physiological and non-physiological conditions [11, 30]. Determining the electrical charge of the membrane as a function of environmental pH, acidic (*C*_TA_) and basic (*C*_TB_) functional group concentrations, and their average association constants with hydrogen (*K*_AH_) or hydroxyl (*K*_BOH_) ions provides a means to monitor changes caused by cancerous transformation [10].

Platinum drugs represent an important class of anticancer compounds. Cisplatin is one of the most widely used in the treatment of many cancers, such as testicular, ovarian, bladder, cervical, head, neck, esophageal, and small-cell lung cancers [14, 23]. Cisplatin reacts with many cellular components that have nucleophilic sites, such as DNA, RNA, proteins, membrane phospholipids, and thiol-containing molecules. Drug reaction with genomic DNA, which is the main event responsible for cisplatin’s anticancer properties, leads to the formation of various adducts that include inter- and intrastrand DNA cross-links and DNA-protein cross-links that inhibit replication and prevent transcription and also affects the translation process [3]. Although DNA binding is generally accepted as the critical pharmacological target of cisplatin-induced cytotoxicity, other cellular targets may also be involved in the cytotoxicity of the drug [2].

**Fig. 1:**
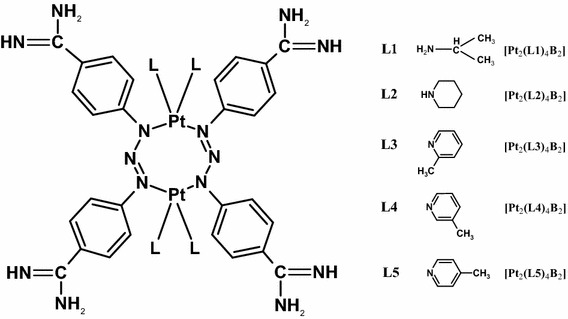
Structure of novel platinum(II) complexes

We aimed to determine the influence of cisplatin and novel dinuclear platinum(II) complexes with the [Pt_2_L_4_B_2_] structure, specifically Pt_2_(isopropylamine)_4_(berenil)_2_, [Pt_2_(L1)_4_B_2_]; Pt_2_(piperazine)_4_ (berenil)_2,_ [Pt_2_(L2)_4_B_2_]; Pt_2_(2-picoline)_4_ (berenil)_2_, [Pt_2_(L3)_4_B_2_]; Pt_2_(3-picoline)_4_ (berenil)_2_, [Pt_2_(L4)_4_B_2_]; and Pt_2_(4-picoline)_4_ (berenil)_2_, [Pt_2_(L5)_4_B_2_] (Fig. 1), on the electrical properties of the membrane, as well as the levels of lipid peroxidation products in human Molt-4 leukemia cells. The quantitative description of cell membrane surface properties may aid in the interpretation and understanding of the processes that take place on biological membrane surfaces during cancer transformation.

## Theory

The model, which has been presented in full detail in previous study [10], assumes that the dependence of surface charge density of cell membrane on pH of electrolyte solution can be described with the help of four equilibria. There are two equilibria of negative groups, with the sodium and hydrogen ions, and two equilibria of the positive groups, with the hydroxide and chloride ions. The H^+^, OH^−^, Na^+^, and Cl^−^ ions are adsorbed at the cell membrane (Molt-4), and the adsorption equilibria (Eqs. 1–4) can by presented in the following form:

1 $${\text{A}}^{ - } + {\text{ H}}^{ + } \Leftrightarrow {\text{AH}}$$

2 $${\text{A}}^{ - } + {\text{ Na}}^{ + } \Leftrightarrow {\text{ANa }}$$

3 $${\text{B}}^{ + } + {\text{ OH}}^{ - } \Leftrightarrow {\text{BOH}}$$

4 $${\text{B}}^{ + } + {\text{ Cl}}^{ - } \Leftrightarrow {\text{BCl}}.$$

The association constants of the H^+^, Na^+^, OH^–^, and Cl^–^ ions with functional groups are expressed by the following equations:

5 $$K_{\rm{AH}} = \frac{{a_{\rm{AH}} }}{{a_{{{\rm A}^{ - } }} \cdot a_{{{\rm H}^{ + } }} }}$$

6 $$K_{\rm{ANa}} = \frac{{a_{\rm{ANa}} }}{{a_{{{\rm A}^{ - } }} \cdot a_{{{\rm Na}^{ + } }} }}$$

7 $$K_{\rm{BOH}} = \frac{{a_{\rm{BOH}} }}{{a_{{{\rm B}^{ + } }} \cdot a_{{{\rm OH}^{ - } }} }}$$

8 $$K_{\rm{BCl}} = \frac{{a_{{\rm BCl}} }}{{a_{{{\rm B}^{ + } }} \cdot a_{{{\rm Cl}^{ - } }} }},$$

where *K*_AH_, *K*_ANa_, *K*_BOH_, and *K*_BCl_ are association constants; $a_{{A^{ - } }}$, $a_{{\rm AH}}$, $a_{{\rm ANa}}$, $a_{{B^{ + } }}$, $a_{{\rm BOH}},$ and $a_{{\rm BCl}}$are surface concentrations of corresponding groups on the membrane surface; and $a_{{H^{ + } }}$, $a_{{{\rm Na}^{ + } }}$, $a_{{{\rm OH}^{ - } }},$ and $a_{{{\rm Cl}^{ - } }}$are corresponding concentrations in solution.

Surface charge density (*δ*) is expressed as follows:

9 $$\delta = (a_{{B^{ + } }} - a_{{A^{ - } }} ) \cdot F,$$

where $F = 96487$ $\left[ \frac{C}{{\rm mol}} \right]$ is the Faraday constant.

Functional group concentration balances are expressed as follows:

10 $$C_{{\rm TA}} = a_{{A^{ - } }} + a_{{\rm AH}} + a_{{\rm ANa}}$$

11 $$C_{{\rm TB}} = a_{{B^{ + } }} + a_{{\rm BOH}} + a_{{\rm BCl}},$$

where $C_{{\rm TA}}$ is the total surface concentration of acidic groups and $C_{{\rm TB}}$ is the total surface concentration of basic groups.

Elimination of $a_{{{\rm A}^{ - } }}$, $a_{{\rm AH}}$, $a_{{{\rm B}^{ + } }}$, and $a_{{\rm BOH}}$ values from above equation yields the following formula:

12 $$\frac{\delta }{F} = \frac{{C_{{\rm TB}} \cdot a_{{H^{ + } }} }}{{a_{{H^{ + } }} (1 + K_{{\rm BCl}} \cdot a_{{Cl^{ - } }} ) + K_{{\rm BOH}} \cdot K_{w} }} - \frac{{C_{{\rm TA}} }}{{K_{{\rm AH}} \cdot a_{{H^{ + } }} + K_{{\rm ANa}} \cdot a_{{{\rm Na}^{ + } }} + 1}}.$$

It is difficult to solve Eq. 12 and determine the $C_{{\rm TA}}$, $C_{{\rm TB}}$, $K_{{\rm AH}},$ and $K_{{\rm BOH}}$ constants. In cases of high or low hydrogen ion concentrations, Eq. 12 can be simplified to linear equations. In the range of high H^+^ concentration, the numerator of each term in Eq. 12 can be divided by the denominator leaving two initial terms only. These operations yield the linear equation in the $a_{{H^{ + } }}$ and $\frac{\delta }{F}a_{{H^{ + } }}$ coordinate system:

13 $$\frac{\delta }{F}a_{{{\rm H}^{ + } }} = \frac{{C_{{\rm TB}} }}{{1 + K_{{\rm BCl}} \cdot a_{{{\rm Cl}^{ - } }} }} \cdot a_{{{\rm H}^{ + } }} - \left( {\frac{{K_{{\rm BOH}} \cdot K_{w} \cdot C_{{\rm TB}} }}{{(1 + K_{{\rm BCl}} \cdot a_{{{\rm Cl}^{ - } }} )^{2} }} + \frac{{C{}_{{\rm TA}}}}{{K_{{\rm AH}} }}} \right).$$

In graphical representation, the slope and the intercept can be easily extracted. At low H^+^ ion concentration, Eq. 12 simplified to

$$\frac{\delta }{F} = \frac{{C_{{\rm TB}} \cdot a_{{H^{ + } }} }}{{K_{{\rm BOH}} \cdot K_{w} + a_{{H^{ + } }} (1 + K_{{\rm BCl}} \cdot a_{{Cl^{ - } }} )}} - \frac{{C_{TA} }}{{K_{ANa} \cdot a_{{Na^{ + } }} + 1 + K_{AH} \cdot a_{{H^{ + } }} }}.$$

The numerator of each term should be divided by the denominator leaving two initial terms only. These operations yield a linear equation in the ${\text{a}}_{{{\text{H}}^{ + } }}^{ - 1}$ and $\frac{{{\delta }}}{\text{F}}{\text{a}}_{{{\text{H}}^{ + } }}^{ - 1}$ coordinate system:

14 $$\frac{\delta }{F}a_{{{\rm H}^{ + } }}^{\_1} = \frac{{ - C_{{\rm TA}} \cdot a_{{{\rm H}^{ + } }}^{ - 1} }}{{1 + K_{{\rm ANa}} \cdot a_{{{\rm Na}^{ + } }} }} + \left( {\frac{{C_{TB} }}{{K_{{\rm BOH}} \cdot K_{w} }} + \frac{{K_{{\rm AH}} \cdot C{}_{TA}}}{{(1 + K_{{\rm ANa}} \cdot a_{{{\rm Na}^{ + } }} )^{2} }}} \right).$$

In graphical representation, the slope and the intercept can be easily extracted.

The coefficients estimated from the linear regression can be used to determine $C_{{\rm TA}}$, $C_{{\rm TB}}$, $K_{{\rm AH}}$, and $K_{{\rm BOH}}$. The points included in the regression must be carefully selected, both in high and low pH ranges. Defining the value of these parameters permits the calculation of the theoretical cell membrane surface charge from Eq. 12 for comparison with experimental data.

## Materials and Methods

### Cell Culture and Treatment

#### Molt-4

Human leukemic T-cell lines Molt-4 (American Type Culture Collection) were maintained in RPMI 1640 medium containing 10 % fetal bovine serum, 50 U/ml penicillin, and 50 μg/ml streptomycin. Cells were cultured in a humidified atmosphere of 5 % CO_2_ at 37 °C. When the cells reached confluency, they were used for assays.

Suspension of cells (1 × 10^6^ cells/ml) in 6 ml of culture medium was incubated with or without the test compounds in cell culture plates.

#### Cell Viability

**Fig. 2:**
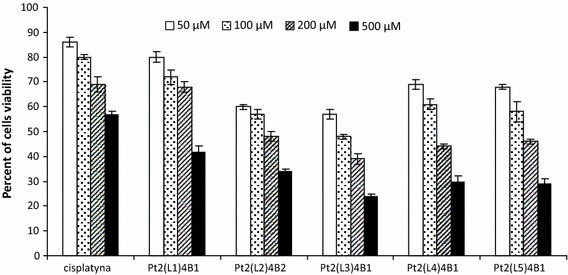
Percent of MOLT-4 leukemic cell viability after 24-h incubation with cisplatin and dinuclear platinum(II) complexes

The platinum(II) complexes [Pt_2_(L1)_4_B_2_–Pt_2_(L5)_4_B_2_] and cisplatin were added to the cultured cells to give a final concentration of 10–500 uM. The control cells were incubated without test compounds. The cells from each cell line were harvested after 24 h of incubation. The MTT test was used for cell viability estimation [18]. Obtained results are shown in Fig. 2.

The IC_50_ values for berenil–platinum(II) complexes were calculated as follows:

Pt_2_(isopropylamine)_4_(berenil)_2_, [Pt_2_(L1)_4_B_2_]—410 μM;

Pt_2_(piperazine)_4_ (berenil)_2_, [Pt_2_(L2)_4_B_2_]—170 μM;

Pt_2_(2-picoline)_4_ (berenil)_2_, [Pt_2_(L3)_4_B_2_]—85 μM;

Pt_2_(3-picoline)_4_ (berenil)_2_, [Pt_2_(L4)_4_B_2_]—150 μM;

Pt_2_(4-picoline)_4_ (berenil)_2_, and [Pt_2_(L5)_4_B_2_]—155 μM;

All experiments were done using complexes at the concentration of 20 μM.

#### Lipid Peroxidation

Lipid peroxidation was estimated by measuring levels of 8-iso-prostaglandine F2α [isoprostanes]. 8-isoPGF2α was assayed according to a modified LC-MS method of Coolen [6]. Briefly, samples were purified using a SEP-PAK C18 column containing octadecylsilyl silica gel. 8-IsoPGF2α was analyzed by HPLC and detected using ESI-MS. Samples were analyzed in the negative multiple reaction mode for MS. Target ions with a m/z 353 → 193 were selected.

#### Electrochemical Method

In order to determine surface charge density of cell membrane, cells were suspended in 0.9 % NaCl and put into the measuring vessel; then, electrophoretic mobility was measured using Zetasizer Nano ZS apparatus (Malvern Instruments). The measurements were carried out as a function of pH.

The surface charge density has been determined using equation *σ* = *ηu*/*d*, where *u* is the electrophoretic mobility, *η* is the viscosity of solution, and *d* is the diffuse layer thickness [16].

The diffuse layer thickness was determined from the formula [1]: $d = \sqrt {\frac{{\varepsilon \cdot \varepsilon_{0} \cdot R \cdot T}}{{2 \cdot F^{2} \cdot I}}}$, where *R* is the gas constant, *T* is the temperature, *F* is the Faraday number, *I* is the ionic strength of 0.9 % NaCl, and εε_o_ is the permeability electric medium.

#### Statistical Analysis

The data obtained in this study are expressed as mean ± SD. The data were analyzed by the use of standard statistical analyses, one-way ANOVA with Scheffe’s F test for multiple comparisons to determine significance between different groups. The values of *p* < 0.05 were considered significant.

## Results and Discussion

Normal cells possess the ability to communicate intra- and intercellularly. Coordination of this information aids in the regulation and integration of cellular functions and growth. However, cancerous cells are not regulated by normal control mechanisms, and they possess other features that distinguish them from normal proliferating cells. For instance, normal cells are well organized in their growth, form strong contacts with their neighbors, and stop growing when they repair an area of injury, due to contact inhibition. By contrast, cancer cells are more easily detached and do not exhibit contact inhibition of their growth. Cancer cells become independent of normal tissue signaling and growth control mechanisms and desynchronized from the rest of the body.

Chemotherapy plays an important role in the treatment of cancer cells. This study was conducted to examine the effects of new chemotherapeutic complexes on the electrical properties and the extent of lipid peroxidation in human leukemia cells

**Fig. 3:**
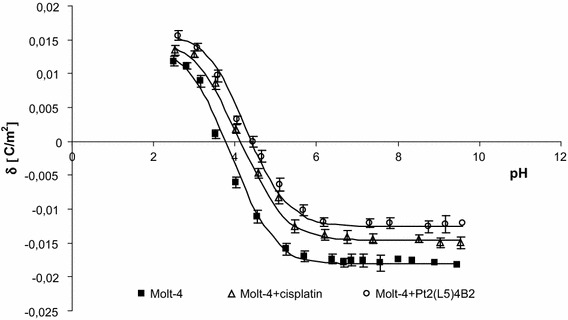
The membrane charge density of Molt-4 leukemia cells with and without treatment with cisplatin and Pt_2_(L5)_4_B_2_. The experimental values are marked by* points* and the theoretical ones by* line*

**Fig. 4:**
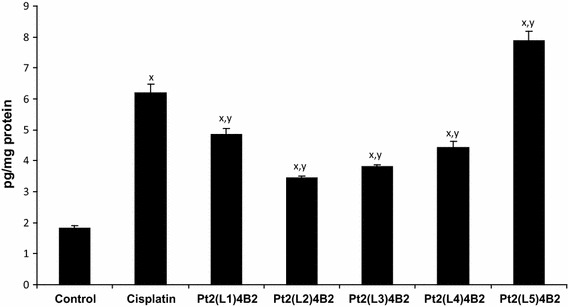
Effect of cisplatin and novel dinuclear platinum(II) complexes on the lipid peroxidation products measured as isoprostanes in Molt-4 leukemia cells. *p* < 0.05, ^x^compared with cancer cell, ^y^compared with cisplatin

The experimental and theoretical surface charge densities of the cell membrane, measured as a function of pH, are presented in Figs. 2, 3, and 4. Experimental measurements are indicated by points, and theoretical values are represented as curves. The surface charge density dependencies of Molt-4 cells on pH produced similarly shaped curves for all studies.

Treatment of Molt-4 cells with cisplatin or novel dinuclear platinum (II) complexes caused a decrease in negative charge at low pH values compared to untreated cells (Fig. 3). The isoelectric point of Molt-4 cell membranes treated with cisplatin or novel dinuclear platinum (II) complexes shifted to higher pH values compared to untreated Molt-4 cell membranes. Probably, it is due to smaller amount of acid groups and greater amount of basic groups of Molt-4 cell membranes treated with cisplatin or novel dinuclear platinum (II) complexes.

Mathematical calculations based on a four-component equilibrium model (presented in Theory), describing the adsorption of electrolyte ions on a cell membrane surface, enabled quantitative evaluation of the membrane parameters. The total concentrations of functional acidic and basic groups on the Molt-4 leukemia cell membrane surfaces and their average association constants with hydrogen and hydroxyl ions were calculated based on Eqs. 13 and 14. Equation 13 was used to calculated the straight-line slope corresponding to the $\frac{{C_{{\rm TB}} }}{{1 + K_{{\rm BCl}} \cdot a_{{Cl^{ - } }} }}$ values and the intersection point with the ordinate axis equal to $- \left( {\frac{{K_{{\rm BOH}} \cdot K_{w} \cdot C_{{\rm TB}} }}{{(1 + K_{BCl} \cdot a_{{Cl^{ - } }} )^{2} }} + \frac{{C{}_{{\rm TA}}}}{{K_{{\rm AH}} }}} \right)$. On the other hand, Eq. 14 used the slope of straight line corresponding to $\frac{{ - C_{{\rm TA}} \cdot a_{{H^{ + } }}^{ - 1} }}{{1 + K_{{\rm ANa}} \cdot a_{{Na^{ + } }} }}$, and the intersection point with the ordinate axis equal to $\left( {\frac{{C_{{\rm TB}} }}{{K_{{\rm BOH}} \cdot K_{w} }} + \frac{{K_{{\rm AH}} \cdot C{}_{{\rm TA}}}}{{(1 + K_{{\rm ANa}} \cdot a_{{{\rm Na}^{ + } }} )^{2} }}} \right)$. The determined $C_{{\rm TA}}$, $C_{{\rm TB}}$, $K_{{\rm AH}},$ and $K_{{\rm BOH}}$ constants were substituted into Eq. 12, yielding the theoretical curve. Typical experimental points and the theoretical curve are presented in Fig. 3. We observed the agreement between the theoretical and experimental surface charge density values.

These results indicated that cisplatin and the novel dinuclear platinum (II) complexes caused a decrease in negative charge at the Molt-4 leukemic cell surface. This finding corresponded with a decreased surface concentration of C_*TA*_ groups. Changes in the functional group composition of the Molt-4 cell membrane could be due to the appearance or disappearance of new functional groups, resulting from the reactions with cisplatin or dinuclear platinum(II) complexes. Treatment with cisplatin or dinuclear platinum(II) complexes increased the association constants of negatively charged K_*AH*_ and decreased the association constants of positively charged K_*BOH*_ groups (Table 1).

**Table 1:** Effects of cisplatin and dinuclear platinum(II) complexes on *C*_TA,_ *C*_TB,_ *K*_AH_, and *K*_BOH_ of Molt-4 leukemia cells

Groups	Parameters			
	*C*_TA_ (10^−7^ mol/m^2^)	*C*_TB_ (10^−7^ mol/m^2^)	*K*_AH_ (m^3^/mol)	*K*_BOH_ (10^7^ m^3^/mol)
MOLT-4	1.90 ± 0.08	1.31 ± 0.06	18.80 ± 1.08	4.97 ± 0.18
MOLT-4 + cisplatin	1.58 ± 0.04^a^	1.48 ± 0.05^a^	22.81 ± 1.10^a^	3.97 ± 0.12^a^
MOLT-4 + [Pt_2_(L1)_4_B_2_]	1.43 ± 0.07^a,b^	1.63 ± 0.06^a,b^	32.80 ± 1.10^a,b^	3.62 ± 0.19^a,b^
MOLT-4 + [Pt_2_(L2)_4_B_2_]	1.45 ± 0.10^a,b^	1.62 ± 0.08^a,b^	35.41 ± 1.11^a,b^	3.54 ± 0.12^a,b^
MOLT-4 + [Pt_2_(L3)_4_B_2_]	1.48 ± 0.10^a^	1.51 ± 0.09^a^	28.89 ± 1.10^a,b^	3.81 ± 0.11^a^
MOLT-4 + [Pt_2_(L4)_4_B_2_]	1.41 ± 0.10^a,b^	1.59 ± 0.10^a^	30.72 ± 1.10^a,b^	3.65 ± 0.11^a,b^
MOLT-4 + [Pt_2_(L5)_4_B_2_]	1.37 ± 0.06^a,b^	1.69 ± 0.05^a,b^	32.46 ± 1.10^a,b^	3.67 ± 0.10^a,b^

*p* < 0.05

^a^compared with cancer cell

^b^compared with cisplatin

Cancer transformation causes modifications in the lipid bilayer membrane [7, 8]. An increase in the phospholipid content has been observed in cancer cells [21, 24]. Increased phospholipid content may result from enhanced cell membrane synthesis related to accelerated neoplasm cell replication [22]. Increased phospholipid levels results in a higher number of functional groups, such as amino, carboxy, and phosphate groups. In acidic medium (low pH), the phospholipid charges are mainly due to amino groups, whereas in basic medium (high pH), it is due to carboxyl and phosphate groups. Increased phospholipids can increase the surface density of negatively charged groups in Molt-4 cell membranes at low pH values and that of positively charged ones at high pH values.

The cell membrane is also affected by sialic acid present in glycolipids and glycoproteins. Sialic acid also may influence the surface concentration of *C*_TA_ and *C*_TB_ groups, as well as the association constants with positive and negative groups during cancer transformation and after drug treatment. The literature indicates that, during cancer transformation, sialic acid content on glycolipids and glycoproteins increases [19, 25, 33]. The decrease of sialic acid on cancer cells after cisplatin or other drug treatments may be associated with an enhanced immune response in the host. It has been suggested that the loss of sialic acid decreases the surface concentration of *C*_TA_ groups and may lead to increased cell deformity and enhanced susceptibility to phagocytosis [20].

Figure 4 shows changes in the level of lipid peroxidation products in Molt-4 cells. Treatment of these cells with cisplatin or the cisplatin complexes caused an increase in isoprostanes level when compared with untreated cells. However, isoprostanes level was the most enhanced in the case of Pt_2_(4-methylpyridine)_4_ (berenil)_2_. Our other studies also show that treatment of these cells with cisplatin or the cisplatin complexes causes an increase in lipid peroxidation products such as HNE and MDA [12].

Membrane lipids and proteins can also be modified by reactive oxygen species (ROS) appearing as the result of cancer transformation, and these ROS provoke lipid peroxidation [15, 28]. As a consequence, phosphatidylserine molecules present in the inner side of the membrane become exposed externally, resulting in additional negatively charged groups [9, 32].

ROS have been reported to play an important role in apoptosis by regulating the activity of certain enzymes involved in the cell death pathway. Many anticancer agents have been found to induce cancer cell apoptosis by increasing ROS [4]. Growing evidence suggests that ROS generation is an important cellular event induced by chemotherapeutic drugs.

Cisplatin, which is an alkylating drug, is also metabolized with increased generation of ROS and disturbances in antioxidant activity [26]. As a consequence, oxidative stress generation is one of the most important mechanisms involved in cisplatin-induced toxicity. This cytotoxic effect is exploited to destroy leukemic blasts and to control leukemia in the second course of chemotherapy [17, 29].

Cisplatinum complexes preferentially attack membrane proteins. Cisplatinum can also interact with phospholipids, but the bonds are relatively weak and reversible [5, 13]. The unstable interaction with phospholipids provokes changes in phospholipid conformation and in the structure and permeability of the membranes. The bond between platinum and monomeric proteins provokes conformational changes and perturbs self-association of the monomers. For example, the binding of cisplatin to the protein actin causes cell death by cross-linking and aggregating monomers, which depolymerizes microfilaments [31].

Proteins bound to DNA react with membrane phospholipids. Their activity in DNA replication, transcription, and recombination is modified by acidic phospholipids [27]. It has been suggested that changes in the membrane composition are connected with changes in cell membrane charge.

In conclusion, the results of the present study demonstrate that novel berenil–platinum(II) complexes more efficiently modify membrane structure and electric properties of cell membrane than cisplatin. Therefore, the constants *C*_TA_, *C*_TB_, *K*_AH_, and *K*_BOH_ may be suitable for monitoring the changes caused by platinum(II) complexes. However, the most potent complex resulting in cellular membrane modifications is Pt_2_(piperazine)_4_(berenil)_2_. Thus, this complex may be valuable for the design of future anticancer drug.
